# Probing the drug delivery strategies in ischemic stroke therapy

**DOI:** 10.1080/10717544.2020.1850918

**Published:** 2020-11-26

**Authors:** Qiong Wu, Rong Yan, Jingjing Sun

**Affiliations:** a Department of Pediatrics, Shengjing Hospital of China Medical University, Shenyang, P. R. China;; b Department of Neurology, Shengjing Hospital of China Medical University, Shenyang, P. R. China

**Keywords:** Ischemic stroke, blood-brain barrier, targeted drug delivery, nanotechnology

## Abstract

Ischemic stroke, which is caused by a sudden clot in the blood vessels, may cause severe brain tissue damage and has become a leading cause of death globally. Currently, thrombolysis is the gold standard primary treatment of ischemic stroke in clinics. However, the short therapeutic window of opportunity limits thrombolysis utility. Secondary cerebral damage caused by stroke is also an urgent problem. In this review, we discuss the present methods of treating ischemic stroke in clinics and their limitations. Various new drug delivery strategies targeting ischemic stroke lesions have also been summarized, including pharmaceutical methods, diagnostic approaches and other routes. These strategies could change the pharmacokinetic behavior, improve targeted delivery or minimize side effects. A better understanding of the novel approaches utilized to facilitate drug delivery in ischemic stroke would improve outcomes.

## 1. Introduction

According to data from the Global Burden of Diseases, Injuries, and Risk Factors Study (GBD), stroke has become more prevalent, with greater mortality and disability worldwide, with noticeable increases in the past two decades (Feigin et al., 2014). A sudden rupture or clot causes a stroke in the cerebral vasculature that prevents blood flow into the brain and leads to severe brain tissue damage (Sims & Muyderman, 2010). Stroke is divided into ischemic stroke (also called "cerebral infarction") and hemorrhagic stroke. Ischemic stroke more commonly presents in clinics, with an incidence greater than 80% (Thom et al., 2006). The brain tissue damage caused by ischemic stroke is a progressive process. First, ischemia causes hypoxia and energy scarcity that initiates a secondary response chain, including the accumulation of reactive oxygen species (ROS), severe inflammatory response and glutamate excitotoxicity (Choi & Rothman, 1990; Rego & Oliveira, 2003). The brain edema, blood-brain barrier (BBB) injury and nervous tissue death induced by the above mutations result in neuronal disorders (Thompson & Ronaldson, 2014). In this review, the conventional treatments against ischemic stroke and their limitations are summarized and discussed, as well as some novel nano-drugs delivery strategies.

## 2. Ischemic stroke drug therapy in the clinic

### 2.1. Thrombolytics

**Figure 1.**
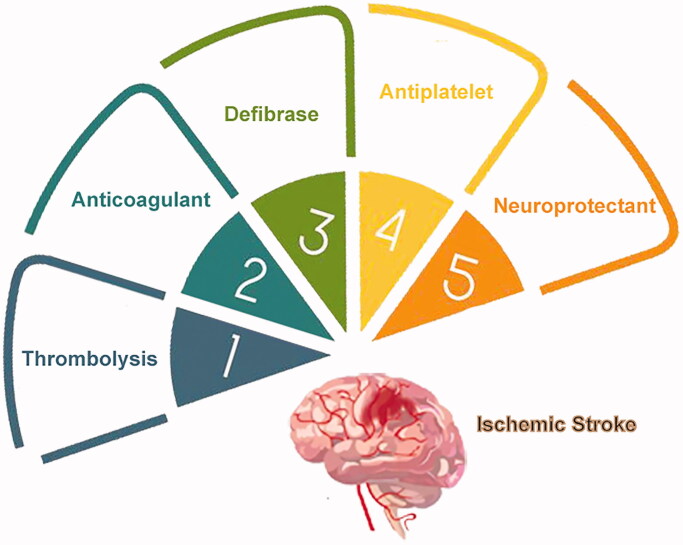
Therapeutic approaches for ischemic stroke.

The therapeutic approaches for ischemic stroke was schematically presented in Figure 1 and the corresponding clinical drugs were summerized in Table 1. Currently, the gold standard treatment of ischemic stroke in clinics is thrombolysis, which lyses fibrin clots in vessels (Powers et al., 2018). Thrombolytics include recombinant tissue plasminogen activator (r-tPA), a second-generation thrombolytics, the only Food and Drug Administration (FDA)-approved pharmacotherapy for management of stroke (Jinatongthai et al., 2017). However, several drawbacks limit thrombolytics use, so only around 7% of patients are eligible for this treatment. The r-tPA therapeutic window of opportunity is restricted to less than 4.5 h from stroke onset, in cases without apparent brain edema or neural tissue destruction. Also, as glycoproteins, thrombolytics have an ultrashort half-life, and therefore frequent or continuous dosing is necessary. Additionally, thrombolytics are nonspecific to the stroke area, leading to some possible serious adverse effects, such as intracranial hemorrhage and heart arrhythmia (Benjamin et al., 2017; Anna et al., 2020). The third-generation thrombolytic, tenecteplase (TNK) is more promising than r-tPA, with better specificity and a longer half-life. However, the therapeutic window of opportunity is still limiting (Coutts et al., 2018).

**Table 1.** This table summarizes clinical therapeutic approaches for treating ischemic stroke.

Drug category	Medicines	Time window	Ref
Thrombolytics	Reteplase (r-tPA)	3–6 h after symptom onset	(Qureshi et al., 2005)
	Tenecteplase (TNK)		(Parsons et al., 2012)
Anticoagulants	Heparin	24 h after symptom onset	(Shrestha et al., 2017)
	warfarin		
Fibrin-Modulators	Defibrase	3–6 h after symptom onset	(Wei et al., 2006)
	Batroxobin		
Antiplatelets	Aspirin	24 h after symptom onset	(Su et al., 2015)
	Clopidogrel		
Neuroprotectants	Edaravone	24 h after symptom onset	(Kikuchi et al., 2013)
	Lovastatin		(Elkind et al., 2008)

### 2.2. Anticoagulants, fibrin modulators and antiplatelet medications

Platelet aggregation, fibrinase levels, and coagulability of blood play an essential role in thrombi formation (Llinas & Caplan, 2001). Hence, doctors only consider anticoagulants, fibrin modulators and antiplatelet medications for patients that do not meet the clinical criteria for treatment with a thrombolytic. Defibrase, heparins, and aspirin are the classical drugs in clinical treatment, which are used alone or in combination according to the state of the stroke patient (Shrestha et al., 2017; Chen et al., 2018). Similar to thrombolytics, these medications are nonspecific and may result in hemorrhage or other hemodynamic response (Cooperative Group for Reassessment of Defibrase, 2005).

### 2.3. Neuroprotectants

**Figure 2.**
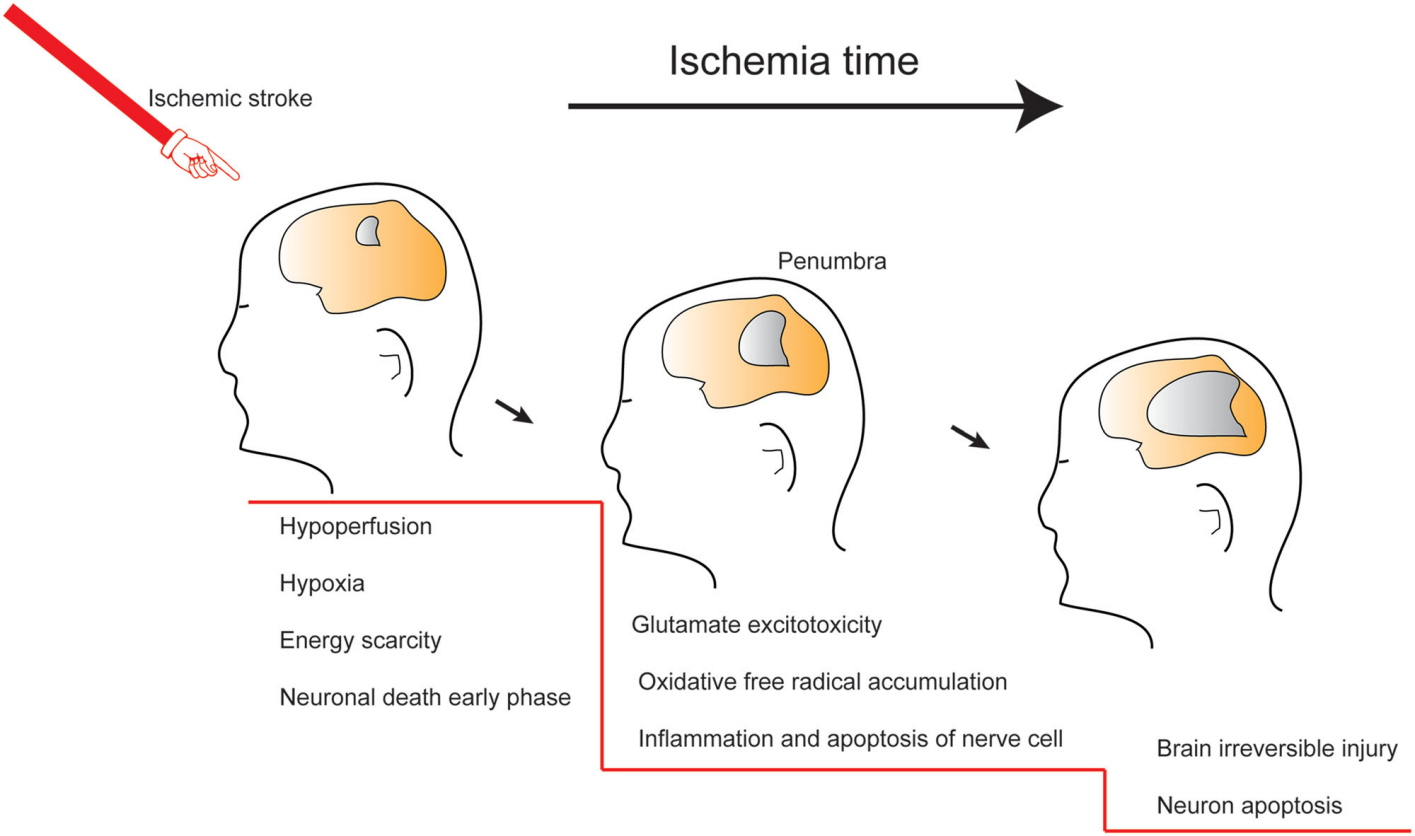
Brain reactions in different phase after stroke onset.

After the onset of cerebral ischemia, a series of cascade reactions occur, including glutamate excitotoxicity, oxidative free radical accumulation, inflammation and apoptosis of nerve cells (Choi & Rothman, 1990; Dirnagl et al., 1999; Brookes et al., 2004; Kim et al., 2006). Additionally, reperfusion of the ischemic injury can cause further damage (Halestrap, 2006). As illustrated schematically in Figure 2, the neuronal damage in the penumbra around the ischemic focus is reversible for a short period if adequate measures are utilized. Neuroprotectants have been developed to prevent and treat different types of secondary damage (Dobkin & Carmichael, 2016; Bernhardt et al., 2017). These medications can fall into several classes: glutamate antagonists, calcium channel blockers, antioxidants, free radical scavengers, and anti-inflammatory and immune factors (Neuhaus et al., 2017). Unfortunately, no country has recommended neuroprotective agents in clinical treatment (Neuroprotection: the end of an era?, 2006; Shi et al., 2018). Disodium 2,4-disulphophenyl-N-tert-butylnitrone (NXY-059) is a novel free radical-trapping agent that showed promising neuroprotective actions in preclinical studies, yet failed in clinical trials (Maples et al., 2001; Lapchak et al., 2002). Several reasons may be responsible for the inadequate clinical translation, including inappropriate animal modeling, individual variation, narrow treatment windows, in effective doses for the brain, and side effects.

## 3. The biological condition of the blood-brain barrier (BBB)

**Figure 3.**
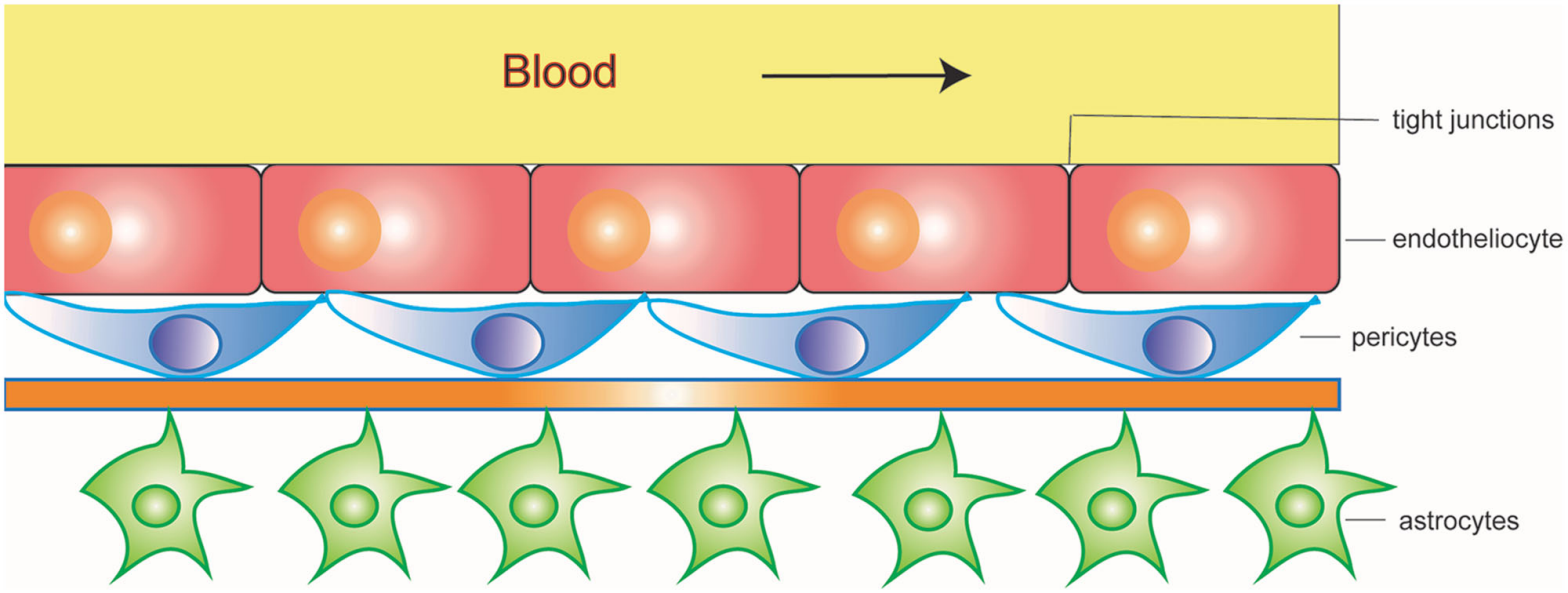
The structure of the blood-brain barrier (BBB).

The blood–brain barrier (BBB) is a significant obstacle that impedes medication transport into the brain (Upadhyay, 2014). The BBB and blood cerebrospinal fluid barrier (BCSFB) are two gateways that regulate substances movement from the blood into the brain. However, the BCSFB plays a lesser role in exchanging substances between the brain and the blood due to its insufficient way and much smaller surface area(Abbott, 2005; Banks, 2009). A series of cells work closely to regulate the BBB's permeability, including endotheliocyte, pericytes, and astrocytes (Figure 3). Tight junctions, adherens junctions, apicobasal polarity, and the luminal surface-bound glycocalyx make up the BBB gating properties (Jain, 2012; Furtado et al., 2018). Efflux transporters such as P-glycoprotein (P-gP), breast cancer–related protein (BCRP), and multidrug resistance-associated proteins (MRPs) also play an important role in the transport of nutrients through the BBB (Zlokovic, 2008). The unique characteristics of the BBB limit the delivery of neuroprotectants into the brain.

**Figure 4.**
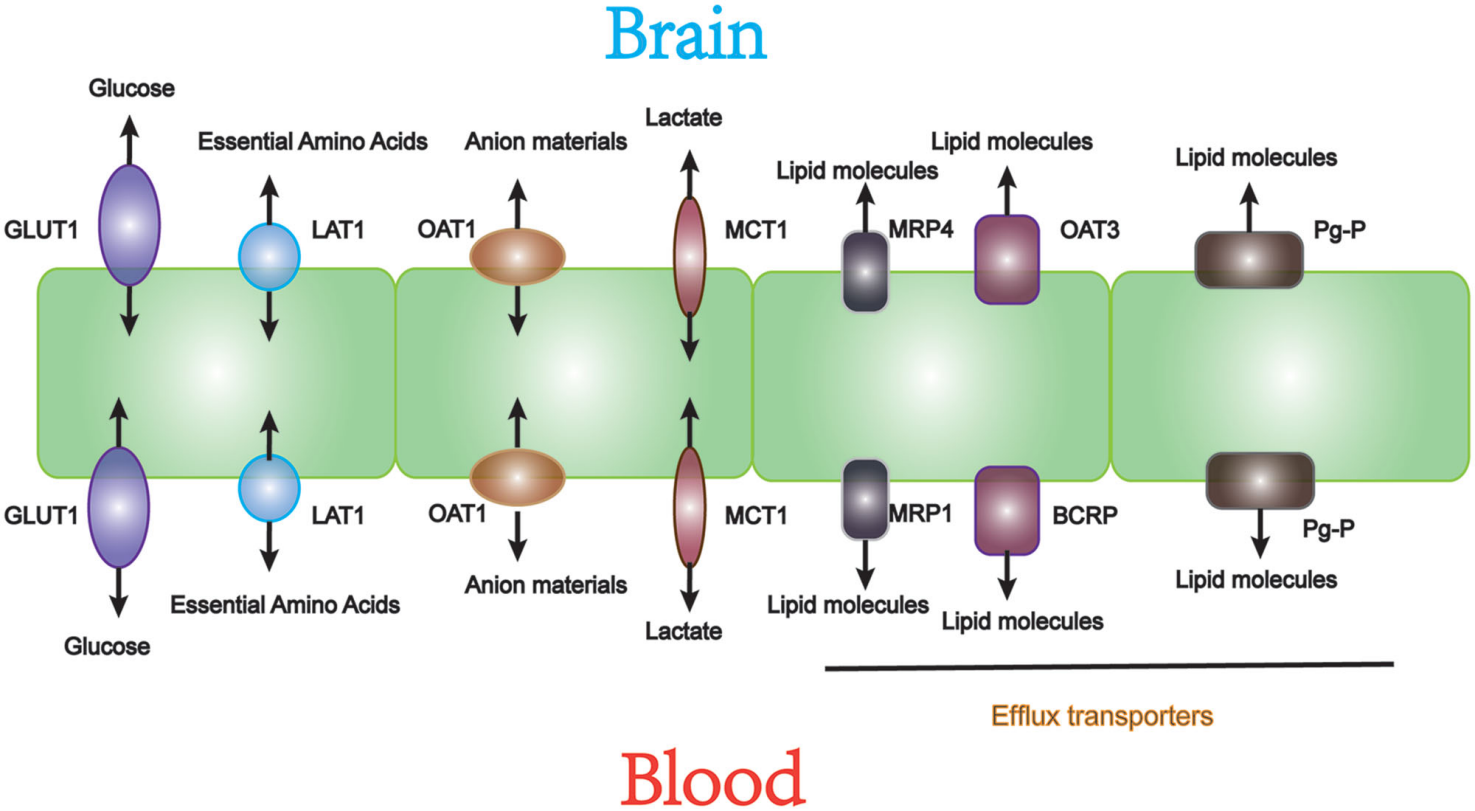
Transporters expressed in the BBB and they facilitate key nutrient molecules exchange between brain and blood.

As shown in Figure 4, specific transporters mediate essential nutrients movement from the blood into the brain. Glucose transporters (GLUTs) transport glucose into cells mainly through sodium-independent diffusion(Simpson et al., 2007). Among them, highly glycosylated GLUT1 is expressed in brain capillaries to ensure the smooth passage of glucose through the BBB (Jurcovicova, 2014). L-type amino acid transporters (L-type amino transporters, LATs) do not depend on sodium or pH, and provide essential amino acids to cells. LAT1 is deeply studied. It is highly expressed in brain microvessel endothelial cells (BMEC). LAT1 has a higher affinity for amino acids than other subtypes of LATs expressed in surrounding tissues. LAT1 plays an important role in the BBB by promoting the entry of nutrients and drugs into the central nervous system (CNS). With a similar structure to the endogenous substrate of LAT1, some drugs can enter the CNS by LAT1 (Gynther et al., 2008). Organic anion transporters (OAT) and organic cation transporters (OCT) are also crucial in the blood-brain material exchange process (Abdullahi et al., 2017). Research has shown that the increased expression of OATP1a4 significantly promoted atorvastatin transport across the BBB, indicating that OATP1a4 may be a new target for CNS drug delivery (Thompson et al., 2014). Other transporters including monocarboxylate transporter 1 (MCT1) and excitatory amino acid transporter 2 (EAAT2), also play essential roles in nutrition exchange between the brain and blood (Furtado et al., 2018). The expression of these transporters in the BBB provides an opportunity for designing targeted-drug delivery to the CNS to treat ischemic stroke.

## 4. Drug delivery strategies targeting ischemic stroke

**Figure 5.**
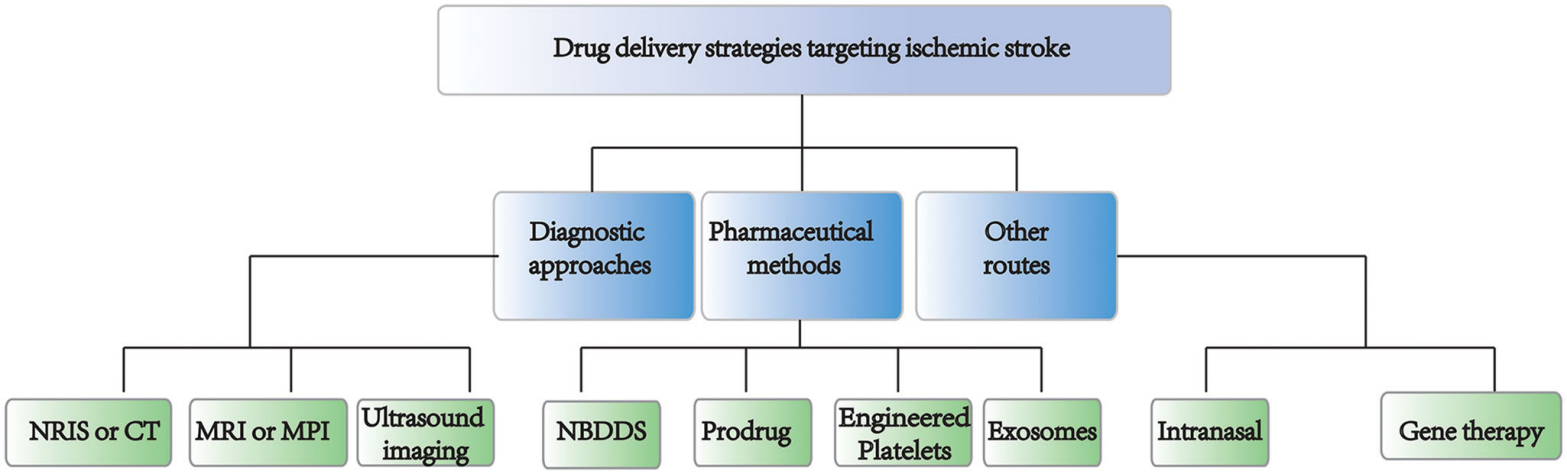
Overview of different strategies for drug delivery targeting ischemic stroke.

Based on clinical treatment's status quo, researchers have made concerted efforts to improve drug delivery to ischemic stroke sites. Many efficient strategies have been developed to solve the limitation of drug treatments (Pardridge, 1997; Gabathuler, 2010; Shcharbina et al., 2013). Briefly, these strategies can be grouped into three categories: pharmaceutical methods, diagnostic approaches, and other routes (Figure 5).

### 4.1. Pharmaceutical methods

In recent years, targeted drug delivery systems have developed rapidly and provided an opportunity to treat ischemic stroke. An ideal drug delivery system that can penetrate the BBB should have the following advantages: release controllability, no damage to the BBB, biodegradability, and brain-targeting. The nanotechnology-based drug delivery system (NBDDS) has become an excellent drug delivery system to the brain due to its small size, large specific surface area, good water solubility, and the ability to be modified. The NBDDS can meet the requirements of an ideal drug delivery system. The prodrug strategy is another commonly used pharmaceutical approach for targeting the brain: prodrugs provide improved stability, selective release, and targeted-delivery. Recently, burgeoning strategies such as cell membrane-encapsulated nanoparticles and exosomes have been employed in ischemic stroke treatment due to their biological targeting properties.

Nanotechnology has been an extensively studied and utilized drug delivery system in the past few decades, including drug delivery in nerve disease (Silva, 2008; Wong et al., 2012). Being nanometers in size and a modifiable structure enable nanotechnologists to carry out multiple desirable functions. These characteristics aid drugs loaded in nanoparticles to possess a longer circulation time and aid their distribution to the brain. There is a great variety of nano-formulations including liposomes, lipid nanoparticles, polymer nanoparticles and micelles. These nanoparticles can be designed and modified to deliver drugs into the brain effectively (Table 2).

#### 4.1.1. Liposomes

Among the variety of drug delivery systems above, the liposome is the most studied one. Liposomes are spherical vesicles prepared by mixing phospholipids and cholesterol in different proportions. The double-layer structure enables them to be loaded with either hydrophilic or hydrophobic drugs and the cell-like membrane structure endows them with low toxicity, good biocompatibility, and prolonged circulation time. In addition, liposomes surfaces are easily modified to achieve targeted-delivery and controlled release. Most importantly, the lipophilicity allows them to enter the brain through the BBB using endocytosis, which has shown to be a promising drug delivery system in treating stroke(Bondì et al., 2012; Joshi et al., 2014). Acetate has been encapsulated within liposomes as an anti-inflammatory drug to attenuate the detrimental inflammation following the onset of an ischemic stroke. The liposomes functioned to extended the half-life and weaken the toxicity of acetate. These liposomes also exhibited increased BBB permeability and significant neuroprotective action through anti-inflammation (So et al., 2019). Liu et al., designed Cytidine-5′-diphosphocholine (CDPC) encapsulated liposomes modified with vascular cell adhesion molecule-1. The CDPC-lipo preferentially accumulated in the ischemic area after overcoming the obstacle of BBB, thus effectively improving the local delivery efficiency of citicoline to the ischemic brain tissue (Liu et al., 2016). It has been confirmed that the neuroprotective effects of liposome-encapsulated withania somnifera, fasudil, tacrolimus or ethylacetate fraction on ischemic stroke significantly enhance due to the increased brain delivery efficiency of liposomes (Fukuta et al., 2015; Ahmad et al., 2016; Fukuta et al., 2016; Singh et al., 2018). A study showed that using fasudil-lipo before tPA could reduce the risk of tPA-induced cerebral hemorrhage as well as prolong the narrow therapeutic time window of tPA. Therefore, taking the advantages of liposomes in brain delivery, it can promote neurorehabilitation and prevent neurodegenerative diseases caused by ischemic stroke. Therefore, liposomes provide an opportunity for effective combined treatment on ischemic stroke (Fukuta et al., 2017). Additionally, liposomes are ideal carriers for antibodies and imaging agents(Sofou & Sgouros, 2008). Mouse paired immunoglobulin-like receptor B (PirB) has proved to be a significant immune action element after cerebral ischemia. Solute PirB (sPirB) ectodomains in polyethylene glycol (PEG)-modified liposomes are used for effective drug delivery to neurons and improve stroke model recovery. sPirB protein may prolong drug circulation and alleviate neuronal apoptosis after exposure to oxygen-glucose deprivation (OGD) by blocking PirB, promoting motor recovery post-stroke. Near-infrared fluorophore 1,1′-dioctadecyl-3,3,3′,3′-tetramethylindotricarbocyanine iodide (DiR) is also encapsulated in immunoliposomes as nanoprobes. Significantly enhanced signal intensities were observed in ischemic hemispheres of the mice's cerebral cortex that had suffered an ischemic stroke after dosing a liposome formulation intravenously into a tail vein. This study indicated sound targeting effects of the liposome formulation to the ischemic cortex (Wang et al., 2018). Generally, liposomes are suitable carriers of anti-inflammatory reagents and can overcome undesired pharmacokinetic behaviors and reduce adverse effects. As a clinically approved formulation, liposomes may become the first nano-formulation candidate of clinical transformation among all the innovative preparations for ischemic stroke treatment.

#### 4.1.2. Solid lipid nanoparticles (SLNs) and nanostructured lipid carriers (NLCs)

Lipid nano-formulations such as solid lipid nanoparticles (SLNs) and nanostructured lipid carriers (NLCs) also help transport drugs into the brain. Lipid nano-formulations are composed of fatty ingredients including fatty acids, long-chain or medium-chain triglycerides, and other lipid materials. Lipophilic drugs are loaded into the lipid nanoparticles, and many possess a high oral bioavailability and long retention time (Pottoo et al., 2020). Additionally, the SLNs or NLCs can be absorbed by apolipoproteins, which mimic lipoproteins, followed by active transport into the brain utilizing receptor-mediated endocytosis (Bramini et al., 2014). Neves et al., developed a resveratrol-loaded SLN with apolipoprotein E (ApoE) on its surface. This functional SLN can be identified by low-density lipoprotein (LDL) receptors, which are overexpressed in the BBB. The in vitro cell permeability assay showed that the SLN functionalized with ApoE could cross the BBB more efficiently than non-functionalized SLN (Neves et al., 2016). Later, Hassanzadeh et al., developed a ferulic acid (FA) loaded NLC, which had improved bioavailability and minimal oxidative stress and neurotoxicity. In ischemic rat models, neurobehavioural deficits were improved significantly (Hassanzadeh et al., 2018). SLNs and NLCs are widely applied in the biomedical field for the treatment of various diseases, especially for brain cancers and neurodegenerative illnesses. Based on their small controllable sizes and the advantage of crossing the BBB, even without any surface modification, SLNs and NLCs are still excellent drug delivery strategies for ischemic stroke therapy. However, low drug loading and drug precipitation of these two dosage forms still need to be resolved urgently.

**Table 2.** Pharmaceutical methods for drug delivery to ischemic stroke.

Drugs applied	Highlights	Effects	Ref
Liposomes			
Cytidine-5′-diphosphocholine	Increased BBB permeability and preferentially accumulated in the ischemic area	Improved the local effective delivered and enhanced neuroprotective effects	(Liu et al., 2016)
Withania somnifera, fasudil, tacrolimus, ethylacetate fraction	Increased BBB permeability	Enhanced brain delivery efficiency and neuroprotective effects	(Fukuta et al., 2015; Ahmad et al., 2016; Fukuta et al., 2016; Singh et al., 2018)
Fasudil, tPA	Increased BBB permeability and combination therapy	Reduce tPA-induced cerebral hemorrhage, prolong the therapeutic time window of Tpa and enhanced neuroprotection	(Fukuta et al., 2017)
Acetate	Improved circulation time, weaken toxicity and increased BBB permeability	Significant neuroprotective action through anti-inflammation	(So et al., 2019)
sPirB	long circulation time and improved BBB permeability	enhanced motor recovery post-stroke	(Wang et al., 2018)
DiR	Significantly enhanced signal intensities in ischemic hemispheres	Targeted imaging of ischemic tissue	(Wang et al., 2018)
Lipid nanoparticles			
Resveratrol	LDL-mediated across BBB	Enhanced neuroprotection	(Neves et al., 2016)
Ferulic acid	Improved bioavailability and minimized oxidative stress	Fewer neurobehavioural deficits	(Hassanzadeh et al., 2018)
Polymer nanoparticles			
NEP1-40	MMP-2-targeted and promoted autocatalysis action of BBB permeability	Improved nerve regeneration after hypoxic-ischemic brain injury	(Han et al., 2016)
Ginsenoside Rg1	TfR-targeted and increased drug delivery into the brain	Enhanced angiogenesis and nerve protection	(Shen et al., 2019)
Rapamycin	CREKA as the targeted head and ROS-responsive drug release	Restored damaged blood-brain barrier and ultimately improved microvascular perfusion	(Lu et al., 2019)
Metal nanoparticles			
Cerium oxide nanoparticles	ROS scavenging and targeting ability to the ischemic brain tissue	Enhanced neuroprotection	(Bao et al., 2018)
Gold nanoparticles	TfR-targeted and ROS scavenging	remarkable neuroprotective effect	(Amani et al., 2019)
Prodrugs			
Valproic acid	Phospholipid (PL) prodrug and reduced the side effect	Enhanced neuroprotection	(Labiner, 2002)
Ketoprofen	Diacetyl glyceride-like structure; Increased oral bioavailability and BBB permeability	Reduced neuroinflammation	(Deguchi et al., 2000)
Engineered platelets			
ZL006e and rtPA	Thrombus targetability	Combination of thrombolysis and neuroprotection	(Xu, Wang, et al., 2019)
Exosomes			
Curcumin	Targeted accumulation without toxicity to the liver or lung	Enhanced anti-inflammation effect	(Zheng et al., 2019)
mir-133b	Efficient delivery and long retention time	nerve remodeling and functional recovery	(Xin et al., 2017)

#### 4.1.3. Polymer nanoparticles

Polymer nanoparticles refer to nano-sized particles composed of natural or synthetic polymer materials. Among them, polylactic acid-glycolic acid copolymer (PLGA) and chitosan are the most widely studied materials and have proven to be effective drug delivery systems (Patel et al., 2012). After loading drugs into nanoparticles, the nanoparticles' surface can be modified and engineered to enhance the targeting property of the NBDDS, increase solubility, extend half-life, reduce immunogenicity, and improve biodistribution. Most studies use PEG coating when using a polymer nanoparticle. The nanoparticles' surface is covered with a PEG lipid layer to reduce nonspecific binding, increase blood half-life, and allow specific ligand binding. Different surface modifications can target the injury site and change the effect based on different treatment purposes. There are many specific receptors expressed in the BBB responsible for transporting large molecules including nutrition transporters, such as insulin receptors, transferrin receptors, low-density lipoprotein receptors, immunoglobulins receptors, and growth factors receptors. Additionally, some molecules are highly expressed in the stroke microenvironment, such as matrix metalloproteinase-2 (MMP-2) and G-protein coupled chemokine receptor-4 (CXCR4). These up-regulated receptors or molecules provide the ability for nanoparticle modification and deliver drugs more efficiently across the BBB into ischemic tissue. Chlorotoxin (CTX) is a substrate of the MMP-2, which is up-regulated in the ischemic microenvironments in brain tissue (Clark et al., 1997; Chang et al., 2003). Han et al., developed a nano-system in which CTX was coated onto PLGA nanoparticles' surface as a targeting ligand. Lexiscan was encapsulated in the nanoparticles to promote an autocatalysis action of BBB permeability. Combining the two approaches to crossing the BBB augmented the engineered nanoparticle's delivery efficiency and could help the loaded NEP1-40 provide a significant therapeutic benefit (Han et al., 2016). Transferrin receptor (TfR) is another frequently-used target ligand that is highly expressed in the cerebral cortex microvessels (Li et al., 2002; Nogueira-Librelotto et al., 2017). Shen et al., conjugated TfR targeted peptide to γ-Poly glutamic acid and ginsenoside Rg1 (Rg1), which was loaded into the obtained TfR-targeted nanoparticles. In vitro brain capillary endothelial cells uptake and in vivo brain tissue exposure of the TfR-targeted nanoparticles exhibited enhanced drug crossing of the BBB and significantly increased drug delivery into the brain (Shen et al., 2019). Similarly, another brain-targeted nanoparticle system was exploited in which organic Angiopep-2 (ANG) was the targeting ligand. Bao et al., prepared a core-shell structured nano-formulation with monodispersed ceria nanoparticles and ANG/PEG as the core and shell, respectively. Edaravone was successfully loaded in the ceria nanoparticles. ANG can specifically bind to the low-density lipoprotein receptor-related protein (LRP) overexpressed on cells that comprise the BBB. Nanoceria is famous for its strong and recyclable ROS scavenging ability due to its shuttling between Ce(^3+^) and Ce(^4+^) oxidation states. Consequently, Ceria and edaravone work together to remove the reactive oxygen species (ROS) caused by the thrombus and protect nerves. In vitro transwell assays confirmed the essential BBB crossing effect of the ANG ligand. ANG-LRP-mediated transcytosis enabled the high accumulation of the ceria and edaravone (Bao et al., 2018). Targeted-ligands make it possible to deliver therapy reagents into the brain and ischemic lesions effectively. In general, polymer nanoparticles have the advantages of good biocompatible and biodegradable, as well as easy surface functionalization. Polymer nanoparticles make it possible to deliver drugs to brain-injured sites of ischemic stroke locally and efficiently.

#### 4.1.4. Micelles

**Figure 6.**
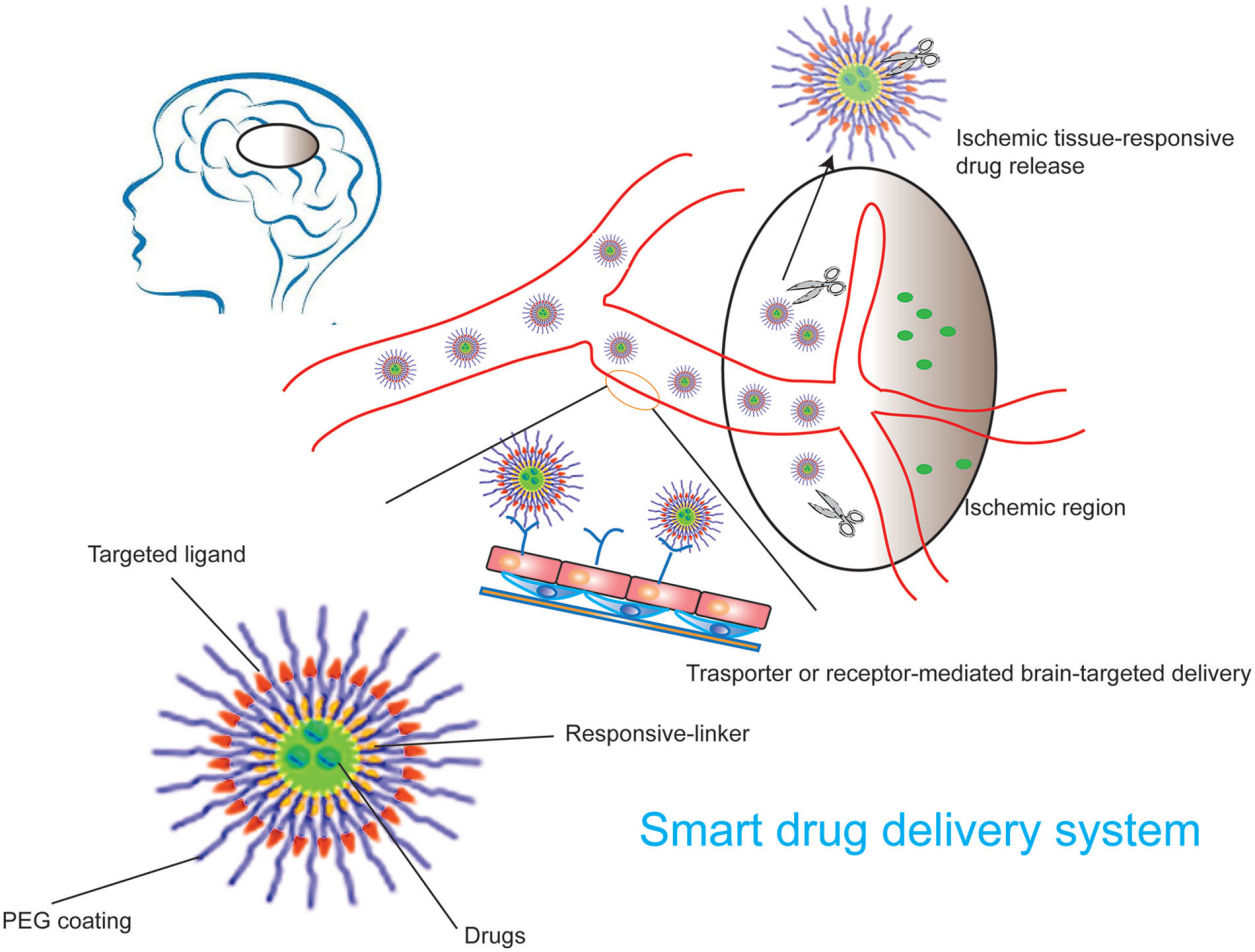
Schematic diagrams of a smart drug delivery system targeting ischemic stroke.

Micelles can also be engineered to release their cargo at the targeted region. For example, a high ROS level is a clear sign of brain lesion tissues after an ischemic stroke and can be used in the stimuli-responsive drug delivery system (Weisenburger-Lile et al., 2019). An innovative and multifunctional micelle system was developed to prevent reperfusion injury via various therapy pathways. Briefly, a ROS-responsive amphiphilic polymer was employed as a carrier, and the fibrin-binding pentapeptide CREKA was linked to the polymer as the targeted head. Rapamycin, a classic mTOR inhibitor and thus anti-inflammatory drug, was encapsulated in the micelles. The NBDDS showed significantly selective release of the drug due to the ROS's different levels between normal tissue and ischemic lesions. In addition, the ROS-responsive polymer also played the role of ROS scavenger. The combination effects of ROS scavenging and mTOR inhibition through rapamycin eased the oxidative stress and decreased neuroinflammation, leading to enhanced neuroprotection and blood perfusion (Lu et al., 2019). Other stimulates also include pH and MMP-2 (Yang et al., 2016; Kumar & Patnaik, 2018). As shown in Figure 6, the selective release of cargos makes it safe and effective to use the NBDDS to deliver drugs to ischemic brain tissues. The advantage of micelles is that hydrophobic drugs encapsulated in the core of micelles can increase its solubility. The smaller particle size is more comfortable to penetrate the BBB, and the surface of micelle is easier to be modified. However, as a brain-targeted drug delivery system, micelles still have many defects in practical applications. First of all, the current selection of targeting molecules mostly uses receptor-mediated targeting strategies, such as polypeptides and monoclonal antibodies, limiting the improvement of targeting capabilities. In addition, the poor stability of the micelles is prone to disintegrate and drug leakage under blood dilution.

#### 4.1.5. Inorganic metal nanoparticles

Inorganic metal nanoparticles have been employed widely in drug delivery. Some researchers focus on their prospects in the treatment of ischemic stroke. Kim CK et al., studied the inherent ROS scavenging ability of cerium oxide nanoparticles in ischemic rat models and found that cerium oxide nanoparticles with a particle size of 3 nm would enter the ischemic brain tissue and reduce the infarct size by half but would not enter healthy brain tissue (Bao et al., 2018). Gold nanoparticles could scavenge ROS in a specific particle size and have shown a remarkable neuroprotective effect. Hamed et al., designed a gold particle-based drug delivery system in which the surface of gold nanoparticles was modified with TfR allowing the gold nanoparticles to target and cross the blood-brain barrier. Gold nanoparticles with a size of 20 nm protect nerves from oxidation, while gold nanoparticles with a size of 5 nm damage nerve cells (Amani et al., 2019). Although inorganic nanoparticles have certain advantages in terms of particle size, synthesis method, surface modification and biocompatibility, the in vivo behavior, toxicity, biodistribution and clearance are also need to investigated before clinical trial studies.

#### 4.1.6. Prodrugs

Structure modification of therapeutic compounds is another commonly used pharmacological approach to improve PK properties and BBB permeability (Pavan et al., 2008). Lipophilic prodrugs have shown great potential in CNS drug delivery. The designed prodrugs cross the BBB in active or passive ways and efficiently release their payloads in specific brain tissues (Markovic et al., 2019). The linker should be enzyme-sensitive or ROS-sensitive or responsive to another specific condition of ischemic lesions. In addition, the lipophilic prodrug may be absorbed with apolipoproteins, which bond to a corresponding receptor on the BBB. Further, the prodrug can be modified with a targeted head, which can be recognized by the BBB or ischemic lesion receptors (Zhao et al., 2014; Wang et al., 2017; Xu, Dong, et al., 2019). The phospholipid (PL) prodrug of valproic acid was designed to penetrate the BBB and release the parent drug using phospholipase A2 (PLA2), reducing the side effects caused by high exposure in the system (Labiner, 2002). Additionally, the anti-inflammatory agent ketoprofen has been coupled with diacetyl glyceride, increasing its oral bioavailibility and BBB permeability (Deguchi et al., 2000).

#### 4.1.7. Engineered platelets

Under physiological conditions, platelets can ensure blood vessel integrity. However, once the blood vessel is injured, activated circulating platelets are recruited to the injured vessel site. This is one of the most critical mechanisms of thrombus formation. At the vessel-wall injury site, aggregated platelets become a major component of the developing thrombus (Nesbitt et al., 2009). Thus, platelet-targeted nanotechnology, such as platelet membrane-encapsulated nanoparticles or platelet biomimetic nano-formulations, has become an effective therapeutic strategy for thrombotic diseases (Wang et al., 2014). Inspired by the role platelets play in thrombus formation, Xu et al., bioengineered a "nanoplatelet" in which neuroprotectant (ZL006e) were loaded in a polymeric nanoparticle core and then encapsulated with a platelet membrane. The platelet membrane was further conjugated with a thrombin-cleavable Tat-peptide-coupled rtPA. Thrombus targetability was enhanced by the platelet's collagen binding feature (Xu, Wang, et al., 2019).

#### 4.1.8. Exosomes

Exosomes are vesicle-like bodies secreted by cells through a series of physiological processes of "endocytosis-fusion-efflux". They have particle sizes ranging from 30–120 nm and are essentially lipid bilayers (Lin et al., 2015). The employments of exosomes in brain drug delivery has been developed in recent decades due to their low immunogenicity and long retention time (Zheng et al., 2019). Remarkably, exosomes also have the ability to cross the BBB. It is well-known that exosomes play a crucial role in intercellular communication in many brain diseases, including the occurrence and development of metastatic brain cancer. According to some previous reports, tumor-derived exosomes can destroy the BBB (Marazioti et al., 2019; Morad et al., 2019; Lu et al., 2020). Therefore, exosomes have great potential in applying drug delivery vehicles for the treatment of brain diseases. If engineered with improved targeting properties for different targeted-ligands, exosomes could be developed into more effective and safe drug delivery systems. Tian et al., isolated the exosome derived from mesenchymal stromal cells (MSC) and conjugated the c(RGDyK) peptide as a targeted-ligand on its surface. Curcumin was used as a model drug with an anti-inflammation effect. The cRGD-exosomes exhibited good targeting capability and no toxicity to the liver or lung(Tian et al., 2018). In addition to being used as a drug delivery carrier, mitochondrial RNA (miRNA) exosomes can be used as markers for the diagnosis and prognosis of ischemic strokes and play an important role in treatment. Xin et al., verified that exosomes could be mediated by mir-133b through MSCs to improve nerve remodeling and functional recovery in ischemic mice models (Xin et al., 2017). However, for these biomimetic nano-formulations, the storage conditions and stability of the preparations still need to be investigated.

### 4.2. Diagnosis strategy

The prognosis of ischemic stroke largely depends on the blood flow reconstruction speed in the ischemic brain tissue; the faster the blood flow reconstruction speed, the better the patient's prognosis. For suspected cases, routine evaluation is required, including diagnostic imaging. The real-time and accurate imaging of cerebral ischemic lesion is essential for diagnosing and safely treating patients with a stroke (Merino & Warach, 2010). Various imaging strategies have been developed, including near-infrared imaging (NRIS), ultrasound imaging, magnetic resonance imaging (MRI), and computed tomography (CT) (Essig et al., 2013; Molad et al., 2017). Nanomaterials also have been used for more efficient and accurate stroke diagnoses. Platelets tend to bind to injured vasculature. This property is the basis for the fabricated platelet (PLT) membrane-derived biomimetic nanobubbles developed for ultrasound monitoring of ischemic stroke lesions. Nanobubbles preferentially accumulate in the stroke's ischemic region and can be monitored by real-time contrast-enhanced ultrasound imaging. This can be beneficial for the proper treatment of strokes (Li et al., 2018). Magnetic particle imaging (MPI) is a new tomographic imaging method with many advantages: it is radiation-free, has a superior temporal resolution, and is easy to operate (Gleich & Weizenecker, 2005; Weizenecker et al., 2009). Peter et al., investigated MPI capabilities in detecting ischemic lesions in a murine model of ischemic stroke. After giving superparamagnetic iron oxide nanoparticles as a contrast agent, the MPI imaging's signal strength was comparable to that of a small animal MRI scanner in detecting ischemic brain regions. However, the acquisition time of MPI imaging was shorter, and it had a higher resolution, which could help precisely predict the patient's condition (Ludewig et al., 2017).

### 4.3. Other routes

#### 4.3.1. Intranasal

Intranasal (IN) is an effective approach to brain drug delivery. This unique delivery route bypasses hepatic metabolism and does not enter the systemic circulation. Without the obstacle of the BBB, the delivery efficiency is also enhanced compared with the intravenous route (Liu et al., 2001; Chapman et al., 2013; Zhao et al., 2017). The exact mechanisms of the nose to brain drug transport have not yet been fully interpreted. However, a prevailing perception is the olfactory pathway. The nasal olfactory epithelium is exposed to a widespread region of the brain through which the drugs can transport into the brain (Lochhead & Thorne, 2012; Pardeshi & Belgamwar, 2013). NR2B9c peptide has been verified as an effective neuroprotectant that blocks N-Methyl-D-aspartate receptors (NMDARs) and downstream neurotoxic signaling pathways. However, as a peptide with a large molecular weight, NR2B9c peptide destabilizes in the blood circulation and has difficulty transporting across the BBB. Additionally, when wheat germ agglutinin (WGA) was added to PEGylation PLGA nanoparticles as a target, the nanoparticles would specifically bind to N-acetyl-D-glucosamine (GlcNAc) and sialic acid residues, which are highly present in the olfactory epithelium. NR2B9c peptide was encapsulated in the targeted nanoparticles and administrated via the intranasal route. In this noninvasive manner, NR2B9c-NPs delivered more NR2B9c into brain tissue and suppressed excitotoxicity, ameliorating neurological function deficits in stroke mouse models (Li et al., 2019).

#### 4.3.2. Gene therapy

With the advancement of molecular biology, new genes are continually being discovered, making gene therapy a promising treatment for stroke worth exploring. Cerebral ischemia can induce the expression of a variety of genes, including some neurotrophic factors (Benraiss et al., 2001). The neurotrophin family includes (1) nerve growth factor (NGF), which can promote the growth and development of nerve cells and prevent nerve cell damage, and (2) brain neurotrophic factor (BDNF). BDNF is highly expressed during cerebral ischemia and hypoxia (Zuccato & Cattaneo, 2009). There are other neurotrophic factors, such as fibroblast growth factor (FGF) and epidermal growth factor (EGF). Gene transfection could be used to cause overexpression of the above genes, thus improving neuroprotection and neurogenesis. The design of gene delivery systems is the key to gene therapy for stroke. Xin et al., designed a ROS-responsive gene delivery system using a ROS sensitive polymer to encapsulate BDNF plasmid, facilitating high-efficiency gene transfection into neural stem cells. The transfected neural stem cells target damaged brain tissue and secrete BDNF to promote nerve regeneration and recovery (Jiang et al., 2019).

## 5. Conclusions and future perspectives

In this review, we described the current clinical situation of ischemic stroke and discussed new approaches to delivering therapy reagents to the injured cerebral issue. As an acute disease, the earlier diagnosis and immediate treatment would be better for the recovery of ischemic stroke. Given the complexity of the disease, drug therapy should not only consider the treatment of stroke, but also consider providing comprehensive protection for the nervous system. However, the limitation of the BBB poses a major challenge for drug delivery against ischemic stroke. According to the biological condition of the BBB, it has shown that burgeoning drug delivery strategies which could potently overcome the BBB embody enormous advantages. Compared with free drugs, a considerable progress has been made in novel drug delivery strategies with improving brain delivery efficiency and drug bioavailability, thereby achieving better efficacy.

Although novel drug delivery strategies have been extensively applied in ischemic stroke, most of the researches are just carried out at the laboratory. Thus, it is crucial how to go out of the predicament in clinical translation of the above innovation preparations. First of all, the lack of biological safety assessment restricts the development of innovation preparations. Secondly, the in vivo molecular mechanism of how novel drug delivery systems break through the BBB has not been clearly addressed, causing the failure of optimizing formulations. Another point that cannot be ignored is that the pathogenesis of ischemic stroke is complex, which involves multiple signaling pathways. The preparation's targeting ability to the injured brain issues is needed to be further investigated. On the complicated pathophysiological condition of the brain, the big successes of these strategies in animals are still difficult to be reproduced in clinical patients. The existence of these problems brings more challenges. A detailed understanding of the pathophysiological mechanisms of ischemic stroke and developing more proper animal models is imperative. Because of the importance of rapid diagnosis to the benefit of early found and therapy of ischemic stroke, there are prospects for the development of theranostics nanoplatform since it combines diagnosis and treatments. In addition, ischemic stroke is closely related to inflammation and oxidative damage. Except for the restored blood flow after thrombotic occlusion, resistance to ischemia-reperfusion injury (such as inflammation and oxidative damage) is essential for a good prognosis. Therefore, it will be a wise choice to design a dual-sensitive drug delivery system that responds to both inflammation and oxidative environment for ischemic stroke in the future. With the extensive study of drug delivery systems and the further exploration of the ischemic stroke mechanism, the novel drug delivery strategies will hold great potential in delivering therapeutics against ischemic stroke.
